# OrthoVenn2: a web server for whole-genome comparison and annotation of orthologous clusters across multiple species

**DOI:** 10.1093/nar/gkz333

**Published:** 2019-05-04

**Authors:** Ling Xu, Zhaobin Dong, Lu Fang, Yongjiang Luo, Zhaoyuan Wei, Hailong Guo, Guoqing Zhang, Yong Q Gu, Devin Coleman-Derr, Qingyou Xia, Yi Wang

**Affiliations:** 1 Biological Science Research Center, Southwest University, Chongqing 400715, China; 2 Department of Plant and Microbial Biology, University of California Berkeley, Berkeley, CA 94710, USA; 3 USDA-ARS, Plant Gene Expression Center, Albany, CA 94706, USA; 4 USDA-ARS, Western Regional Research Center, Crop Improvement and Genetics Research Unit, Albany, CA 94706, USA

## Abstract

OrthoVenn is a powerful web platform for the comparison and analysis of whole-genome orthologous clusters. Here we present an updated version, OrthoVenn2, which provides new features that facilitate the comparative analysis of orthologous clusters among up to 12 species. Additionally, this update offers improvements to data visualization and interpretation, including an occurrence pattern table for interrogating the overlap of each orthologous group for the queried species. Within the occurrence table, the functional annotations and summaries of the disjunctions and intersections of clusters between the chosen species can be displayed through an interactive Venn diagram. To facilitate a broader range of comparisons, a larger number of species, including vertebrates, metazoa, protists, fungi, plants and bacteria, have been added in OrthoVenn2. Finally, a stand-alone version is available to perform large dataset comparisons and to visualize results locally without limitation of species number. In summary, OrthoVenn2 is an efficient and user-friendly web server freely accessible at https://orthovenn2.bioinfotoolkits.net.

## INTRODUCTION

Homologous genes can be divided into two main classes: orthologs and paralogs. Orthologous genes originate from a common ancestor during specification events (1), and are usually syntenic between close-related species. Paralogs also share a common ancestor, but arise from sequence duplication events within a species, and often show limited synteny and more speciation-related divergence. If orthologous genes in multiple species show high sequence similarity, the likelihood that they may continue to perform similar biological functions (2). On the other hand, orthologs with sequences that show greater divergence from other species are more likely to perform distinct functions (2). In recent decades, identifying orthologous genes and ascertaining the degree of similarity between them are two important steps in comparative genomics studies to understand the evolution of genes and genomes (3). New innovations in sequencing technologies have rapidly generated vast amounts of genome-wide sequence data across the tree of life (2,4,5), and there is a growing need for tools that enable orthologous gene identification and the ability to explore their function and evolution across phylogenetic space.

Two fundamental methods for identifying orthologous clusters have been developed. The first is tree-based, and several tools such as software PhyloTreePruner (6) and TreeFam (7) employ this strategy. The second is graph-based, and this methodology is found in tools including PanOCT (8), OrthoFinder (9), OrthoMCL (10), COCO-CL (11), OrthoDB (12), OMA (13) and Roary (14). The tree-based and graph-based algorithms differ in many respects and each of them have their own advantage, but current tree-based algorithms are generally computationally more expensive to construct for large numbers of genes and genomes, which makes this type of approach less applicable than the graph-based approach for orthologous analysis on large datasets with large evolutionary distances (3). For these reasons, both OrthoVenn and OrthoVenn2 employed a graph-based method which has been demonstrated utility in automating and handling large datasets (3).

Most applications for orthologous gene identification and comparison were developed for use on Linux-based systems, including OrthAgogue (15), Ortholog-Finder (16), Orthograph (17), PorthoMCL (18) and ProteinOrtho (19). While this feature contributes to their power, speed and versatility, it also limits the pool of potential users to researchers who have specific familiarity and knowledge with the Linux OS. To address this issue, a growing number of web-based orthology detection and comparison tools have been developed recently, such as OrthoInspector (20), Family-Companion (21), Orthonome (5), PhosphOrtholog (4), Hieranoid (22) and MorFeus (23). However, the majority of these tools lack the ability to visualize orthologous clusters, and the few that have this capability, such as ORCAN (24), contain relatively rudimentary visualization capabilities to support the comparisons of large datasets from multiple genomes.

Previously, we published a high-speed web-server based tool, OrthoVenn, which was published in the 2015 *Nucleic Acids Research* web server issue (25). This tool has been used and cited in a growing body of research (26), and thousands of users from more than sixty countries have used it to analyze their datasets. The utility of OrthoVenn has been cited more than 150 times since its release.

Here, we present an update to the OrthoVenn tool based on requests from users and the broader community of comparative genomics researchers. In this update, we have implemented new features to allow users to perform whole genome comparisons for up to twelve species of bacteria, fungi, protists and metazoa (an increase from the six in the previous version). As a result of the relatively larger genome size of plants and vertebrates as compared with the other types of organisms listed above, users can choose up to a maximum of eight species for genomic comparisons when working within these two eukaryotic groups. To improve speed, the alignment between related species within clades has already been calculated and included in the web version of OrthoVenn2. The pre-calculation and alignment between distant species would substantially increase the storage amount in our server. Due to the storage requirements and computational efficiency, the current web version of OrthoVenn compares species within clades, and comparisons between distant species (such as the species from different kingdoms) are not supported. To help facilitate cross-kingdom comparisons, and more generally for processing and visualization of larger numbers of species, users can either upload their own user-generated clustering dataset from other softwares as an input to OrthoVenn, or download the stand-alone version for use on their own server. In addition, we introduced significant enhancements to the data visualization and interpretation capabilities of OrthoVenn2. OrthoVenn2 continues to be open to all users and freely available as a web service at https://orthovenn2.bioinfotoolkits.net. The input data for OrthoVenn2 is a protein sequence in fasta format and the output is an interactive occurrence pattern table and Venn diagram with additional layers of information and downloadable content. To demonstrate its utility, we randomly chose eight *Streptomyces* species for comparative analysis of orthologous genes with OrthoVenn2. The results are discussed below in the ‘sample and result analysis’ section.

## DATASET

We downloaded protein sequence from the Ensembl database (release January 2019) and incorporated them into OrthoVenn2 following our previous pipeline (25). This dataset includes 142 vertebrates, 71 metazoa, 65 protists, 94 fungi, 57 plants and 111 bacteria species. The total number of protein sequences present in OrthoVenn2 is 8 858 566. In total, the protein database in OrthoVenn2 is four times the size of the previous version. The annotation of protein clusters was performed through DIAMOND analysis using the non-redundant protein database in UniProt (release January 2019) as described previously (25,27).

## GENOME COMPARISON IN MULTIPLE SPECIES

**Figure 1.**
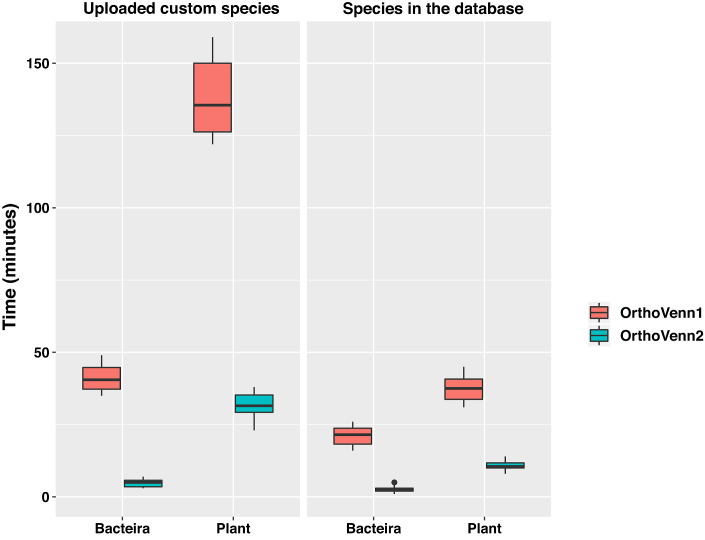
Speed comparison of OrthoVenn1 and OrthoVenn2. Boxplots of median time lapse per job for OrthoVenn1 (red) or OrthoVenn2 (Green). The boxplots indicate the median (central line), the first and third quartiles (upper and lower box bounds) and the minimum and maximum value (lower and upper whiskers).

The previous version of OrthoVenn only supports whole-genome comparisons for up to six species due to limitations in computational capacity and visualization methods. In OrthoVenn, we used the most popular heuristic best-match method available at the time (3) from OrthoMCL (10) to identify orthologous genes based on conservation (25). In this update, we provided new features that allows users to compare orthologous genes for more than six species (a maximum of twelve for bacteria, fungi and protists, whereas up to eight for plant and vertebrate, due to their large genome size could hinder computational efficiency of our server). First, we upgraded our web-server to have an increased computational capacity to support additional comparisons. Briefly, the prior server for Orthovenn1 had 16 core processors and 96G memory, while the new Orthovenn2 server harbors 64 core processors and 512G memory. Second, OrthoVenn2 uses DIAMOND (v0.9.24) instead of BLASTP or UBLAST to perform the all-against-all protein sequence comparison as DIAMOND has been shown to be 20 000 times faster than BLASTX and 1000 times faster than UBLAST without any significant compromise in output reliability or value (28). To test the speed improvement after update, we chose six plant and six bacteria species within our database and performed two separate orthologous clustering with both Orthovenn1 and Orthovenn2 to compare their respective speeds. Additionally, we uploaded six custom species of plant and bacteria to perform clustering with Orthovenn1 and Orthovenn2, separately. The results indicate that OrthoVenn2 is at least ten times faster than OrthoVenn1 (Figure 1). As a complement, a stand-alone version has been also developed that can process multiple species without limitations on capacity. Finally, the accuracy of OrthoVenn2 was compared with other orthology inference methods using the benchmark service from the QfO community (29). OrthoVenn2 showed accuracy similar to InParanoidCore, OMA Groups 2.0, eggNOG and SonicParanoid (fast) (Supplementary Figure S1).

## TOOLS FOR VISUALIZING RESULT SETS

**Figure 2.**
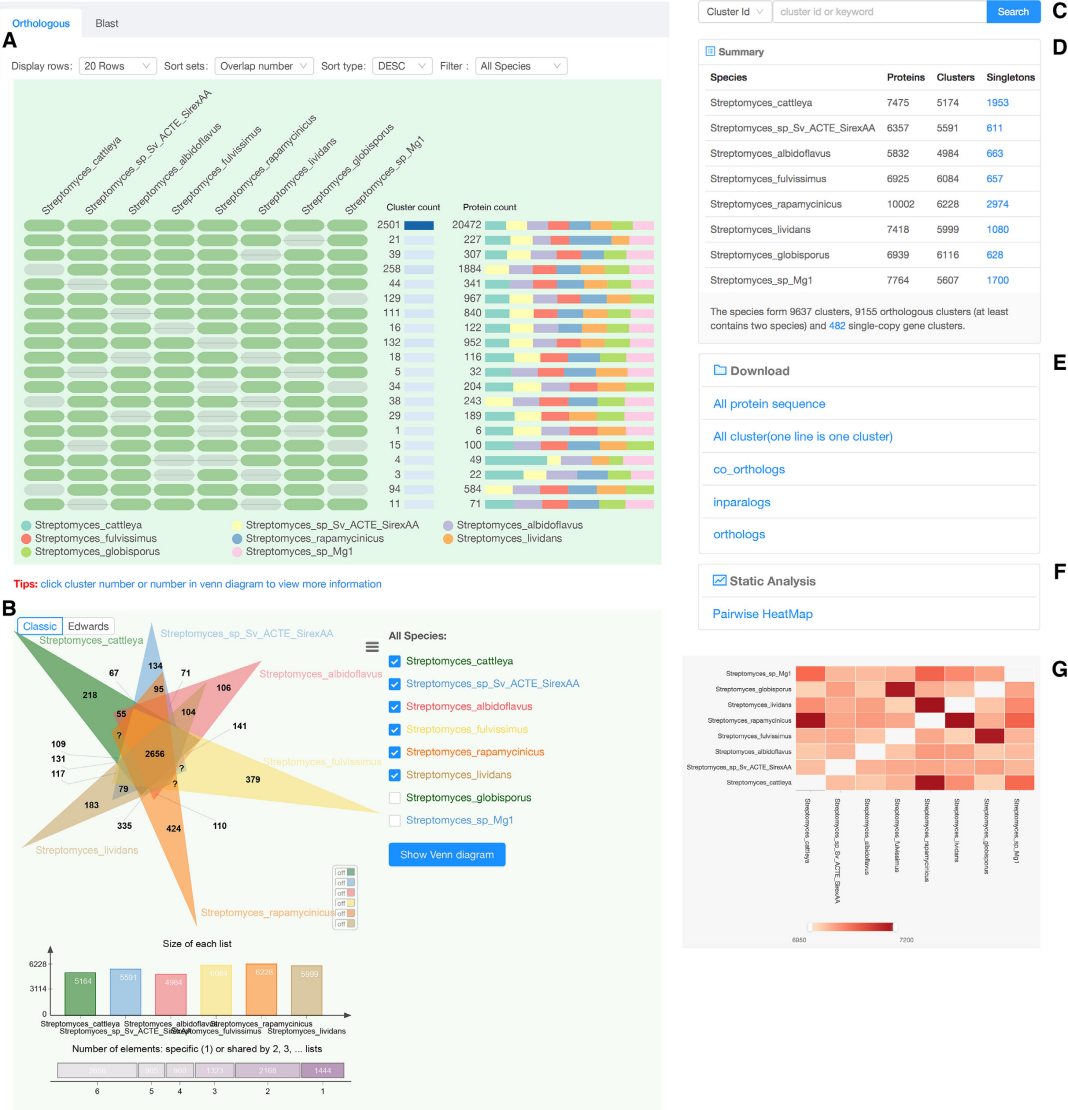
A result page of Orthovenn2. (**A**) The occurrence table shows the occurrence pattern of shared orthologous groups among *Streptomyces cattleya, Streptomyces sp. Sv ACTE SirexAA, Streptomyces albidoflavus, Streptomyces fulvissimus, Streptomyces rapamycinicus, Streptomyces lividans, Streptomyces globisporus* and *Streptomyces sp. Mg1*. The pattern to the left indicates which species are in the clusters, cluster count is the number of clusters shared between species, and protein count is the number of protein members in the shared clusters. (**B**) Venn diagram displays the distribution of shared orthologous clusters among the first six species. (**C**) Keyword and cluster ID search for specific clusters in the results. (**D**) Counts of clusters in each genome. The singleton at the top right describes the genes for which no orthologs could be found in other species; single copy gene clusters at the bottom indicate the clusters that contain single copy gene in each species. (**E**) Download links for computed datasets. (**F**) A link to the pairwise heatmap of overlapping cluster numbers between pair-wise genomes. (**G**) The pairwise heatmap of overlapping cluster numbers appears in the pop-up. The heatmap of overlapping cluster numbers between each pair of genomes. Each cell indicates the overlap cluster numbers between each pair of species. The overlapping cluster numbers refers to the cluster numbers that were shared between species. The overlapping cluster numbers would appear when users hovering cursor over each heatmap cell.

To display multiple comparison results more effectively, OrthoVenn2 uses an occurrence cluster table to display the orthologous cluster groups for multiple species (Figure 2A) with associated informative Venn diagrams (Figure 2B). This clustering result is summarized as a cell graph in which each row represents an ortholog cluster group and each column indicates a species. A green cell indicates the presence of a cluster group in the corresponding species, and a gray bar represents the absence of a cluster group in that species (Figure 2A). There are other visualization options that users can select before downloading the figure, including changes to cell color, height, width, and font size by clicking the icon in the upper right corner. The table can be redrawn with several user-defined sorting schemes, including sorting by protein number, overlap count, or cluster count in descending (‘DESC’) or ascending (‘ASC’) order (Figure 2A). Overlaying the cursor on each cell will display the species identity associated with the current cell (Figure 2A). A stacked bar plot at right displays the cumulative number of protein sequences present in the cluster group for each species (Figure 2A).

OrthoVenn2 also provides a search function to retrieve specific clusters from the archived cluster results with a keyword or cluster ID (Figure 2C). The total number of shared clusters for each pair of species are summarized in a table (Figure 2D). Links are also provided for users to download the computed datasets (Figure 2E), and a ‘Pairwise HeatMap’ button will render a heatmap to visualize the overlapping cluster numbers for the working species in a pairwise fashion (Figure 2F). The overlapping cluster numbers are indicated through a color gradient with user-defined minimum and maximum thresholds. Overlapping clusters less than the minimum threshold are ignored and indicated by blank cells within the figure (Figure 2G). Additionally, we offer a BLAST tool on a second tab to allow users to input their own FASTA-format sequences to compare against the output clusters (Figure 2A).

**Figure 3.**
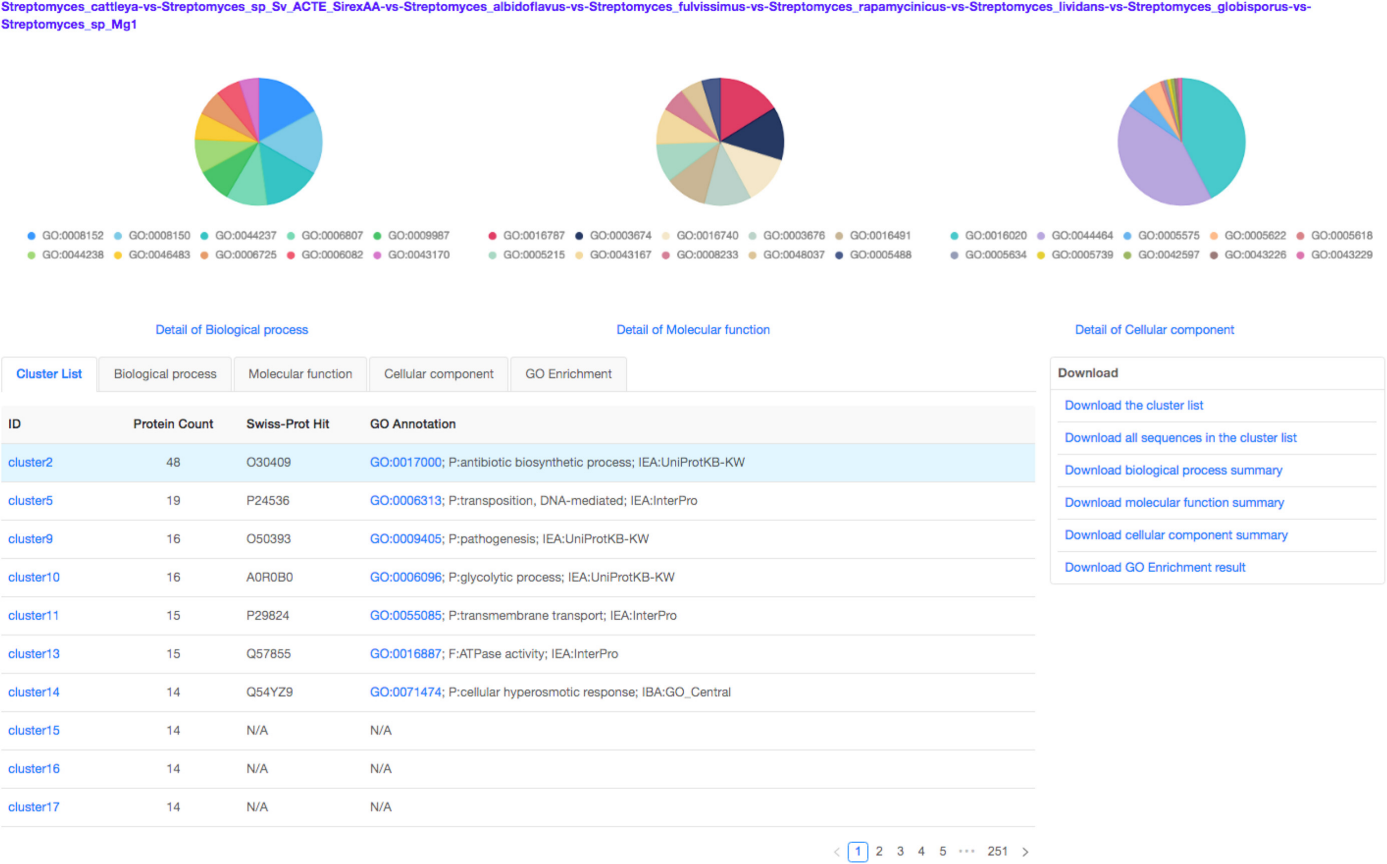
Distribution of GO terms for core orthologous gene clusters of eight *Streptomyces* species.

**Figure 4.**
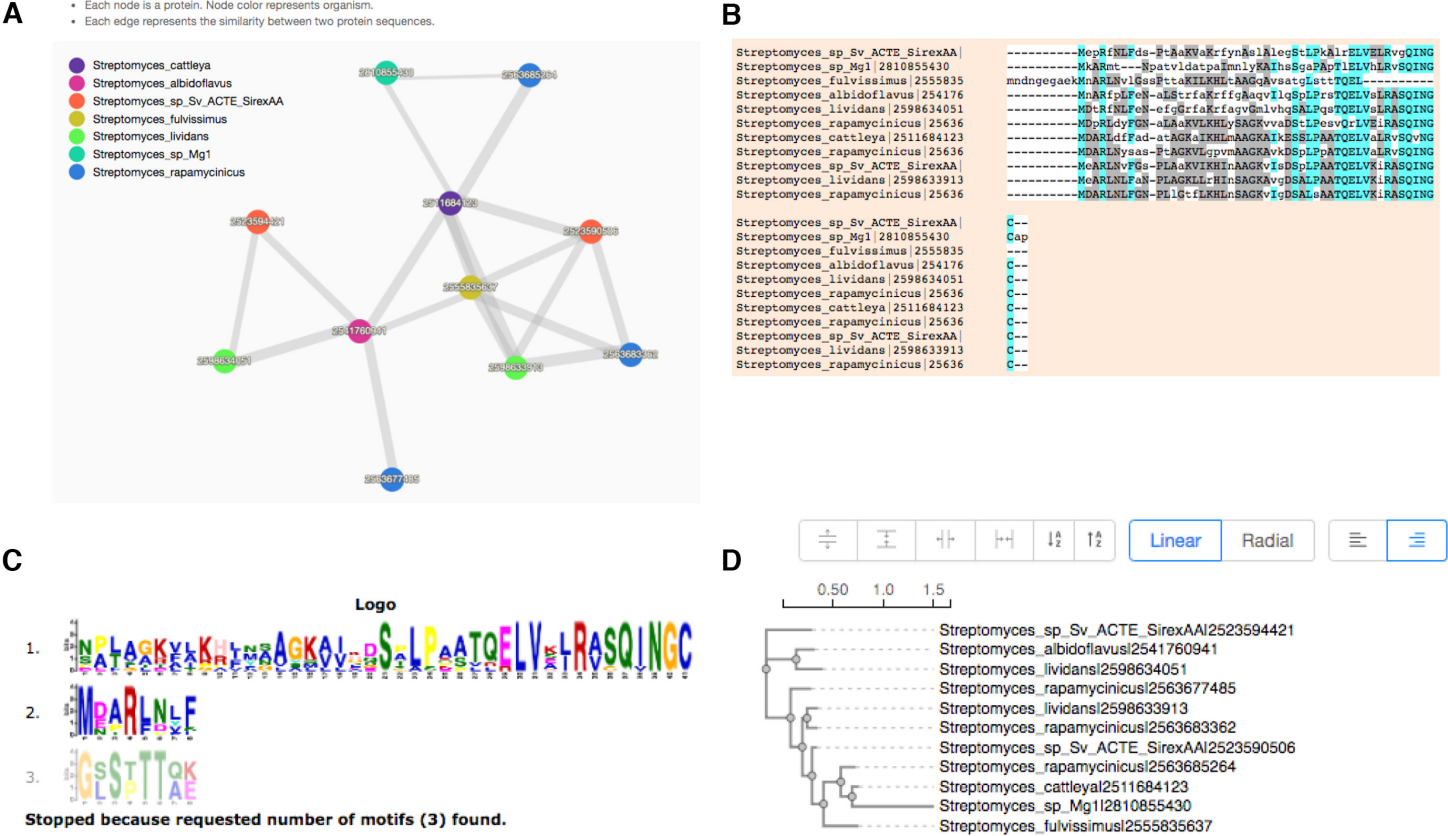
The annotation of cluster65 using multiple methods. (**A**) The network of proteins within cluster65. (**B**) Multiple sequence alignment for proteins in cluster65. (**C**) Motifs in the protein sequences in cluster65. (**D**) Phylogenetic tree for the proteins within cluster65.

Finally, to render a Venn diagram in ‘Classic’ or ‘Edwards’ format for up to six species from the occurrence table, the user may select the checkboxes for individual species names located below the occurrence table. By default, a Venn diagram is automatically drawn for two to six of the species present in the analysis (Figure 2B). The color of each species in the Venn diagram can be adjusted by selecting the species name (Figure 2B). Additionally, a bar plot below the Venn diagram shows the number of clusters found in each species (Figure 2B, bottom). One can download these figures by selecting the icon above and to the right of the Venn diagram. Selecting the number found within each cell of the Venn diagram or the cluster number in the occurrence table will produce a new page showing the functional information associated with the chosen cluster group (Figure 3). It should be noted that the functional annotation can only be performed for up to eight species. Three pie plots are used to visualize the proportion of GO terms for the three main functional categories: biological processes, molecular functions and cellular components within the chosen orthologous group. The corresponding numeric information can be found on the ‘Cluster list’ page. The network for each selected cluster ID can also be generated (Figure 4A). To download the fasta file for the current cluster, the user can select the ‘fasta’ link. An alignment figure can be generated by clicking on the ‘Multiple Sequence Alignment’ button (Figure 4B). Finally, OrthoVenn2 also incorporates the software ‘Multiple Em for Motif Elicitation’ to perform a motif analysis (Figure 4C) and a phylogenetic tree for users (Figure 4D).

## SAMPLE AND RESULTS ANALYSIS

To demonstrate the utility of OrthoVenn2, we applied OrthoVenn2 clustering to eight randomly picked *Streptomyces* species. Pairwise genome comparisons were performed with the following parameters: e-value 1e-5 and an inflation value 1.5. The results can be found at https://orthovenn2.bioinfotoolkits.net/task/result/8576ec1a59ad3d78b2680ab78b181191. Briefly, the analysis identified 9,637 orthologous clusters, which includes 2501 core genome orthologs (Figure 2B). The number of core genome orthologs is less than the number (3,096) reported in a study in which five *Streptomyces* genomes were compared (30). To address the discrepancy, we hypothesized that different *Streptomyces* species sets harbor different core genome orthologs. To test our hypothesis, we downloaded these five genomes of *Streptomyces* species from NCBI http://www.ncbi.nlm.nih.gov/genome/browse/, predicted genes with prodigal v2.6.3 (31) and performed the genome comparison using OrthoVenn2 with threshold e-value 1e-5 and inflation 1.5. We identified 8694 orthologous clusters in total with 3188 core orthologous gene set https://orthovenn2.bioinfotoolkits.net/task/result/ae808cda97a1384ca03d200fdc4571c0. These results are consistent with the previous study. The number of predicted orthologous groups ranges from 8,341–9,209 using *E*-values of 10, 1, 0.1, 0.01, 10–3, 10–4, 10–5, 10–6, 10–7, 10–8, 10–9 and 10–10 and inflation indexes of 1.0, 1.5 and 2.0 (30). Our observations demonstrated that OrthoVenn2 is a reliable and user-friendly tool to perform genome orthologous comparison.

## ClusterVenn IMPROVEMENT

As there are many methods available for ortholog clustering, for example, OrthoMCL can generate cluster file. In order to provide users a function of viewing the shared clusters between species, we developed a tool named ClusterVenn to visualize this cluster file in OrthoVenn1. This early version was limited to analysis of orthologous clusters for a maximum of six species due to known visualization constraints of Venn diagrams with more than six categories (32). In this update, we used the occurrence table to display the occurrence of cluster groups between species, allowing users to upload and compare clusters without limitations of species number. Simultaneously, users can choose up to six species in the occurrence table to display the intersection and disjunction relationship between species with a Venn diagram. Cluster files from most available orthologous identification tools are compatible and visualizable with OrthoVenn2.

## STAND-ALONE VERSION

In response to users’ requests, we generated a stand-alone version of OrthoVenn2 for large datasets comparisons. Our tool is merged with Docker technology to build reproducible and convenient types of workflows. Docker is an open source project and platform for building, shipping and running any app, enabling the widespread distribution of applications (https://docs.docker.com). The release of OrthoVenn2 as a Docker provides an isolated and self-contained package without the need to install dependencies and change environmental settings. This feature increases its reusability and reproducibility while simplifying its ease of use. The installation and usage instructions are available at https://orthovenn2.bioinfotoolkits.net/download. Users can input their own fasta file without limitation of species number. In almost all other respects, the usage is the same as that for the web server, including data analysis and visualization.

## FUTURE PLAN

OrthoVenn2 is an open-source web server that identifies and compares genome orthologs from different species. We upgraded our server capacity to process larger datasets and offered improvements to data visualization and interpretation. However, OrthoVenn2 might have some backlog in term of data analysis and interpretation. Currently, OrthoVenn2 only takes protein sequence data as input. We are working to improve our computational capacity to allow users to upload both protein and the genome sequences. Input of genome sequences could allow our tool to predict genes and perform protein translation. The protein sequence will be clustered and annotated in our current version. We also aim to continue to improve the visualization and annotation of orthologous groups.

Some of the orthologous regions show collinearity characteristic (33,34). Comparative analysis of the collinearity for these segments is important to understand the genome rearrangements and evolution. We wish to add the collinearity comparison for our orthologous in our future version.

## Supplementary Material

gkz333_Supplemental_File

## Notes

Present address: Ling Xu, Department of Plant and Microbial Biology, University of California Berkeley, Berkeley, CA 94710, USA.
