# Supplementary material for: OrthoVenn2: a web server for whole-genome comparison and annotation of orthologous clusters across multiple species
Source: Nucleic Acids Res. 2019 May 4;47(W1):W52–8. doi: 10.1093/nar/gkz333 (PMC6602458; doi:10.1093/nar/gkz333)
Supplement: gkz333_Supplemental_File [file gkz333_supplemental_file.pptx]

## Slide 1
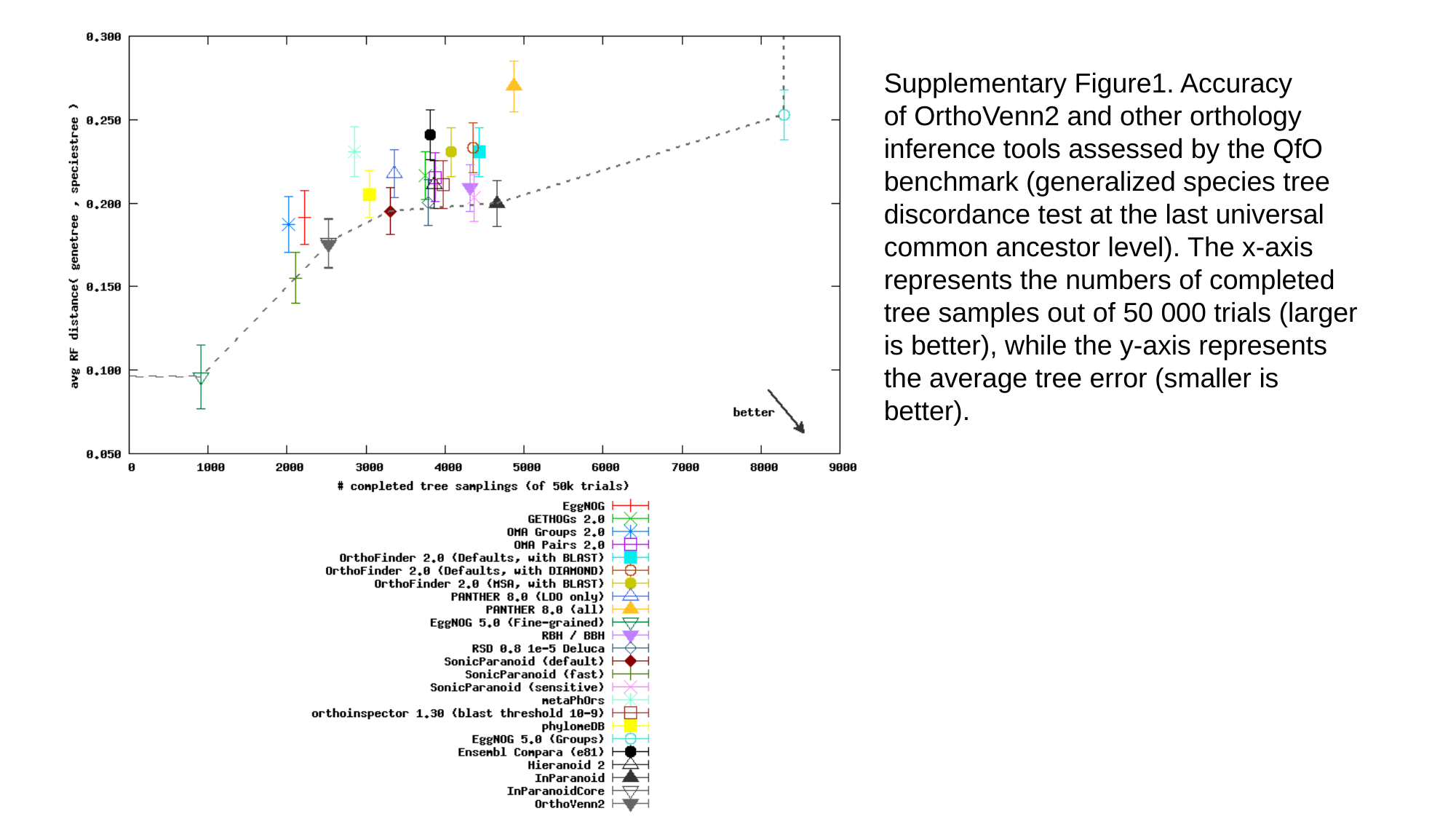

Supplementary Figure1. Accuracy of OrthoVenn2 and other orthology inference tools assessed by the QfO benchmark (generalized species tree discordance test at the last universal common ancestor level). The x-axis represents the numbers of completed tree samples out of 50 000 trials (larger is better), while the y-axis represents the average tree error (smaller is better).
